# Experiences and perspectives of older patients with a return visit to the emergency department within 30 days: patient journey mapping

**DOI:** 10.1007/s41999-021-00581-6

**Published:** 2021-11-10

**Authors:** Bo Schouten, Babiche E. J. M. Driesen, Hanneke Merten, Brigitte H. C. M. Burger, Mariëlle G. Hartjes, Prabath W. B. Nanayakkara, Cordula Wagner

**Affiliations:** 1 Department of Public and Occupational Health, Amsterdam UMC, Vrije Universiteit Amsterdam, Amsterdam Public Health Research Institute, De Boelelaan 1117, P/O Box 7057, 1007 MB Amsterdam, The Netherlands; 2 Department of Emergency Medicine, Amsterdam UMC, Vrije Universiteit Amsterdam, Amsterdam Public Health Research Institute, De Boelelaan 1117, Amsterdam, The Netherlands; 3 Section General and Acute Internal Medicine, Department of Internal Medicine, Amsterdam UMC, Vrije Universiteit Amsterdam, Amsterdam Public Health Research Institute, De Boelelaan 1117, Amsterdam, The Netherlands; 4 Netherlands Institute for Health Services Research (NIVEL), Utrecht, The Netherlands

**Keywords:** Emergency/acute medicine, Older patients/aged, Patient journey mapping, Patient perspective, Patient-centered care

## Abstract

**Purpose:**

Up to 22% of older patients who visit the emergency department (ED) have a return visit within 30 days. To achieve patient-centered care for this group at the ED it is important to involve the patient perspective and strive to provide the best possible experience. The aim of this study was to gain insight into the experiences and perspectives of older patients from initial to return ED visit by mapping their patient journey.

**Methods:**

We performed a qualitative patient journey study with 13 patients of 70 years and older with a return ED visit within 30 days who presented at the Amsterdam UMC, a Dutch academic hospital. We used semi-structured interviews focusing on the patient experience during their journey and developed a conceptual framework for coding.

**Results:**

Our sample consisted of 13 older patients with an average age of 80 years, and 62% of them were males. The framework contained a timeline of the patient journey with five chronological main themes, complemented with an ‘experience’ theme, these were divided into 34 subthemes. Health status, social system, contact with the general practitioner, aftercare, discharge and expectations were the five main themes. The experiences regarding these themes differed greatly between patients. The two most prominent subthemes were waiting time and discharge communication, which were mostly related to a negative experience.

**Conclusions:**

This study provides insight into the experiences and perspectives of older patients from initial to return ED visit. The two major findings were that lack of clarity regarding waiting times and suboptimal discharge communication contributed to negative experiences. Recommendations regarding waiting time (i.e. a two-hour time out at the ED), and discharge communication (i.e. checklist for discharge) could contribute to a positive ED experience and thereby potentially improve patient-centered care.

**Supplementary Information:**

The online version contains supplementary material available at 10.1007/s41999-021-00581-6.

## Key summary points

**Aim:**

To achieve patient-centered care for older patients at the emergency department (ED) it is important to include their perspective and experience, and this can be done through the patient journey method.

**Findings:**

By mapping the patient journey, we found that waiting times and suboptimal discharge communication are almost always related to a negative experience for older patients.

**Message:**

The novelty of this study lies within the qualitative patient journey method, which allowed us to include the voice of the patient in issues that have been previously described (i.e. waiting times and discharge communication). We believe this can guide towards patient-centered improvement initiatives that can contribute to a positive ED experience in the future, for example a time-out at the ED and a discharge check list

**Supplementary Information:**

The online version contains supplementary material available at 10.1007/s41999-021-00581-6.

## Introduction

Over the past decade the pressure on emergency care has been increasing to levels where the demand exceeds the available resources [1]. This phenomenon, also known as emergency department (ED) crowding, is a major challenge worldwide for acute healthcare provision. It has negative consequences for the efficiency, quality, and safety of emergency care [2, 3]. ED crowding is partially caused by the growing number of older people with complex medical and social situations who visit the ED [2]. Globally, older patients account for up to 30% of all ED visits, and this proportion will continue to increase, more than can be expected based on demographic changes alone[4–9]. This group of older patients is increasing in age, frailty, multi-morbidity, and polypharmacy, causing emergency care to be highly complex [10, 11]. Furthermore, up to 22% of the older patients have an unplanned return visit to the ED within 30 days of the initial ED visit [8, 9, 12–14]. A return ED visit may indicate a potential fragile state a patient is in, resulting in potential negative consequences for the patient (i.e. increased risk for functional decline and mortality[14]), and for the healthcare system (i.e. higher healthcare costs[15]).

With the increasingly complex ED care (many tests, treatment options and clinical pathways), the preferences and needs of patients are not always discussed and taken into account, which can contribute to a negative ED experience [16]. For example, Han et al. showed that older patients in Taiwan with an early ED return visit often felt neglected and not being taken seriously during their initial ED visit [17]. Though return visits related to relapse or worsening of existing medical problems are not always potentially preventable (i.e. exacerbation COPD) [18], it is important to endeavor giving patients the best possible experience during all ED visits.

Patient experience is an important element to achieve patient-centered care [19]. Patient-centeredness has been identified as one of the quality of care domains by the Institute of Medicine Committee on Quality of Health Care. They define patient-centered care as follows: “responsive to the patient’s preferences, values, and needs, with compassion and empathy”, with the goal to customize care to each individual patient, instead of the patient to care [20]. Previous research has shown that patient-centered care can lead to multiple positive outcomes, i.e. better health outcomes, increased patient satisfaction, and decreased healthcare utilization and costs [21–24].

Patient experience is determined by multiple facets that range from healthcare professional-patient communication to convenience factors such as the availability of meals [25, 26]. Not every facet of patient experience will have an (or similar) effect on clinical outcomes; however, in order to pinpoint novel areas of improvement it is important to include and study the perspectives and experiences of patients.

Patient journey mapping is a method developed to measure and visualize a patient’s perspective and experiences throughout their care journey [27]. Mapping the patient journey of older patients with a return visit within 30 days to the ED may shed light on issues that are important from a patient perspective and could lead to new initiatives to improve the experience of older patients. Therefore, the aim of this study was to provide insight into the experiences and perspectives of older patients from their initial to their return ED visit (within 30 days) by mapping their patient journey and to provide a visual overview of this journey.

## Methods

### Study design and setting

We conducted a qualitative patient journey study at the ED of the Amsterdam UMC, location VUmc, using semi-structured interviews with patients. This qualitative study was part of a larger study (PRISMA, investigating return visits) at the ED that ran from February to November 2018. All patients included in the patient journey study were also participating in the PRISMA study [28, 29], the aim of the PRISMA study was to identify root causes contributing to an ED (return) visit in older patients. Patients for the patient journey study were included from May to November 2018.

This study has been granted approval by the ethics committee of Amsterdam UMC, location VUmc (2017.579).

### Participants and data collection

Patients of 70 years and older with a return visit to the ED within 30 days were eligible for inclusion. The return visit did not have to be related to the initial ED visit. Exclusion criteria were as follows: patients younger than 70 years of age; not living independently; not able to give informed consent; critical care room presentations; scheduled return visits; unable to conduct the interview within two weeks after the return visit.

The inclusion was based on convenience sampling, meaning that patients were included from Monday till Friday during daytime hours (10 am to 8 pm). Patients eligible for inclusion were approached face-to-face by a researcher from the PRISMA-study, who assessed whether the patients were also interested to participate in the patient journey study. When interested in participation they were asked to sign an informed consent form, which were stored safely at the Amsterdam UMC, location VUmc. A researcher contacted the patient the day of the return visit (face-to-face or by telephone) to provide additional information regarding the study and to schedule a date for the interview. The interview was preferably conducted within one week, but no longer than two weeks after the ED return visit.

To capture and visualize the patient’s perspective and experiences throughout their care journey, we conducted semi-structured interviews with patients within two weeks after their return visits [27]. We designed a topic list for the semi-structured interviews, based on the literature [30–35] and the expertise of the research team (consisting of medical doctors, psychologists, and a sociologist), see online resource 1. The topic list covered the total patient journey from before the initial ED visit until after the ED return visit, focusing on the multiple facets of patient experience [25, 26]. We pilot tested the topic list in the first four interviews, after which we concluded that the list was comprehensive and that revisions were not necessary. Interviews were conducted at the patient’s place of residence or, when admitted after their ED return visit, at the hospital or supportive care facility. When available and approved by the patient, their partner could contribute during the interview. However, questions were always primarily directed at the patient. No repeat interviews were carried out. The duration of the interview varied between patients, but took 90 min on average. Interviews were recorded with a voice recorder, and audio files were stored digitally at the Amsterdam UMC location VUmc to which only the researchers had access. During the interview, field notes were taken to supplement the transcripts in the analysis process. Interviews were conducted by two researchers (BB: May–July and MH: September–November), both female medical students (BB in her final study year, and MH fourth year), supervised by a medical doctor and psychologist. Both interviewers gained experience regarding interview- and counselling techniques during their medical education. Patients were included until data saturation was reached. This was evaluated after each interview, by analyzing patterns and themes in the new data and comparing this to the themes that had emerged from the existing data.

### Data analysis

Patient characteristics and follow-up variables (see Table 1 and online resource 2) of included patients were derived from the PRISMA study database, with consent from the patients. Interviews were transcribed by the initial interviewers, using the audio files. Transcripts and/or results were not returned to the patients for review and feedback.

**Table 1:** Characteristics per patient

Interview nr	1	2	3	4	5	6	7	8	9	10	11	12	13
Age in years (5 year intervals)	75–79	> 85	75–79	70–74	80–84	80–84	75–79	80–84	> 85	75–79	80–84	70–74	70–74
Sex	Male	Male	Female	Male	Male	Male	Female	Male	Female	Male	Female	Male	Female
Living with partner^a^	No	Yes	Yes	Yes	Yes	No	No	Yes	No	Yes	No	Yes	Yes
Home care^a^	No	No	No	No	No	Yes	No	No	Yes	No	No	Yes	No
Medication use nr.^a^	< 5	< 5	≥ 5	≥ 5	≥ 5	≥ 5	≥ 5	< 5	≥ 5	≥ 5	< 5	≥ 5	< 5
GP visits^a,b^	2	6	1	4	1	12	2	0	4	2	3	2	5
ED visits^a,b^	1	1	1	3	5	1	3	3	1	2	5	3	0
Referral													
Initial	GP	GP	Self	Specialist	Self	Specialist	Specialist	GP	GP	GP	GP	Specialist	GP
Return	Specialist	GP	Specialist	Specialist	Specialist	Specialist	GP	Specialist	Self	Self	GP	Specialist	Specialist
Transport													
Initial	Ambulance	Ambulance	Own	Own	Own	Own	Ambulance	Own	Ambulance	Ambulance	Own	Own	Own
Return	Own	Ambulance	Own	Own	Own	Own	Ambulance	Own	Ambulance	Own	Own	Own	Own
Triage code^c^													
Initial	< 10 min	< 10 min	> 1 h	< 10 min	> 1 h	> 1 h	< 10 min	< 1 h	< 10 min	Directly	< 10 min	< 1 h	< 10 min
Return	< 1 h	< 10 min	< 10 min	< 1 h	< 10 min	< 10 min	< 10 min	< 1 h	< 1 h	> 1 h	< 10 min	< 10 min	< 1 h
APOP score^d^													
Decline	21	42	27	19	63	25	37	20	69	22	25	11	14
Mortality	12	24	3	8	40	13	3	4	3	12	1	7	3
Complaint initial and return visit related^e^	Yes	Yes	Yes	No	Yes	Yes	Yes	Yes	Yes	Yes	Yes	Yes	Yes
Discharge from ED													
Initial	Original place of residence	AMU VUmc	Original place of residence	Original place of residence	Original place of residence	Original place of residence	Admission other hospital	Original place of residence	Original place of residence	Original place of residence	Original place of residence	Ward Vumc	Ward VUmc
Return	AMU VUmc	AMU VUmc	Original place of residence	Original place of residence	Original place of residence	AMU VUmc	AMU VUmc	Original place of residence	Ward Vumc	Ward Vumc	Original place of residence	Ward Vumc	Ward Vumc
Deceased^a,f^	No	No	No	No	No	Yes	Yes	No	No	No	No	No	No

[59]

*GP* general practitioner, *ED* emergency department, *AMU* acute medical unit, *VUmc* VU university medical center

^a^Return visit as target

^b^In the previous year

^c^The time the patient has to be seen in

^d^Acute Presenting Older Patient (APOP) score [59]. Calculated based on age, gender, arrival by ambulance, fall- related visit, ADL support, hospital admission during the last 6 months, and a cognition test. The scores indicate the risk of functional decline and mortality in the next three months. Score ranges from 0–100; a higher score represents a higher risk

^e^As judged by the patient

^f^Within six months after return visit

Data analysis consisted of an inductive approach of direct content analysis, in order to identify categories and map the patient journey [36]. Researcher BS (psychologist, female) open coded four interviews, and developed a first draft of the framework based on the open themes. This first draft of the framework was used to code three interviews and based on this, complemented with new themes. Consensus was reached on the second draft of the framework with researcher HM (psychologist, female). After this, researcher BD (medical doctor, female) independently coded the same four interviews with this version of the framework, and after consensus with BS new themes were added to the framework again. This process of independently coding, fine-tuning, and consensus between BS and BD was repeated multiple times to finalize the framework. In the final version of our framework we reached thematic saturation, therefore making it suitable for the coding of all interviews.

All interviews were coded independently by one psychologist (BS) and one medical doctor (BD), to account for differences in interpretation. This was followed by an extensive consensus process, in order to maximize reliability and credibility of our results. If there were discrepancies in codes between BS and BD, these were solved in the consensus process, resulting in 100% agreement regarding the final codes. Both the subject/phase of the ED visit and the experience (positive/negative) were coded, see the results section for comprehensive information on the framework and coding process. Coding was performed in Atlas.ti7, and descriptive statistics were performed using IBM SPSS Statistics version 21.

## Results

Data saturation was reached after 14 interviews, as no new information, themes, and patterns emerged during the interim analyses. Out of these 14, one interview (former I4) was eventually excluded after thorough consideration and consultation with the research team, because of the complexity of the journey with many ED visits which made it impossible to code the journey in a reliable way. The exclusion did not affect data saturation. Therefore, the final analysis and development of the framework was based on the 13 eligible interviews.

Table 1 shows the characteristics per patient and online resource 2 shows the patient and ED visits characteristics on group level. Our sample consisted of 13 older patients with an average age of 80 years, 62% of whom were males.

### Conceptual framework

The basis of our conceptual framework was in accordance with the timeline of the patient journey and an additional experience theme, which also corresponds to the chronological order of the topic list during the interviews. The phases of the journey and, therefore, the five chronological main themes of our conceptual framework were the following: before initial ED visit, initial ED visit, between ED visits, return ED visit, and after return ED visit. The ‘experience’ theme was complementary to the five chronological main themes. The five chronological main themes and the complementary ‘experience’ theme consisted of a total of 34 subthemes, which emerged from the data. Multiple subthemes could be assigned to the same text fragment. In almost all text fragments the experience theme was coded with another theme. To illustrate this, on a text fragment where a patient voiced negative experiences with waiting times during their initial visit the following subthemes were coded: ‘before initial visit-waiting times’ and ‘negative experience’. We used this to identify which moments in the journey were related to either positive or negative experiences, and also to capture an overall ED experience.

**Fig. 1:**
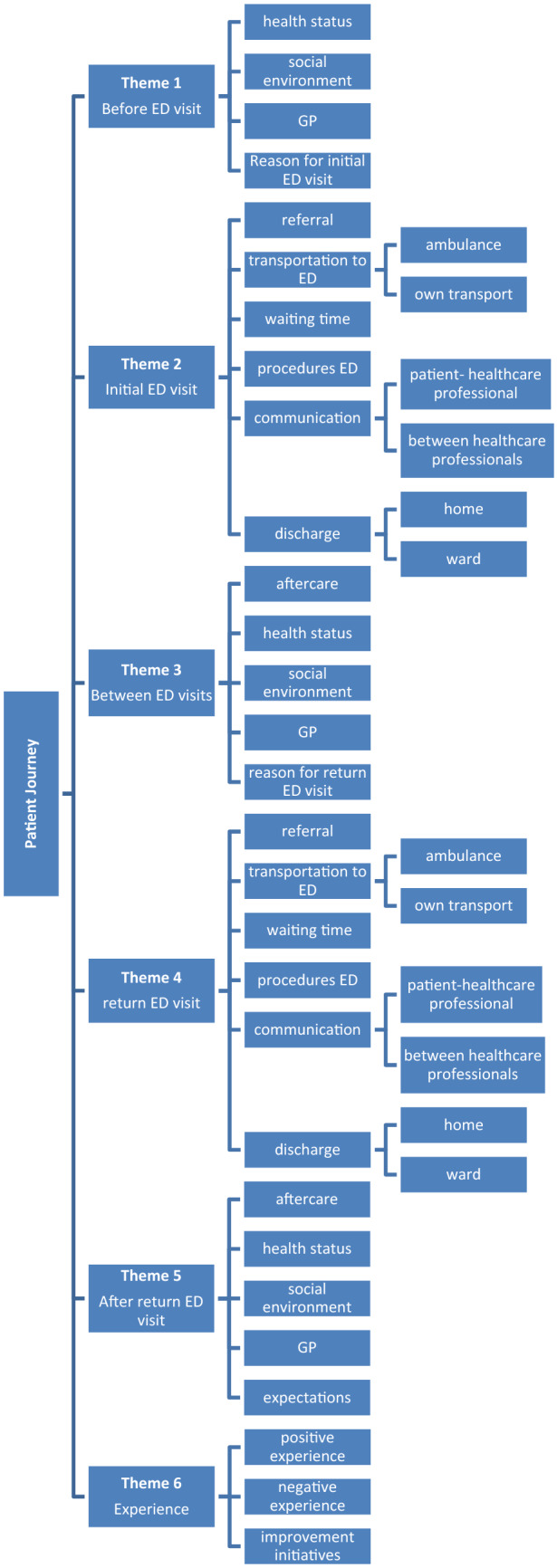
Conceptual frame work—coding tree

Figure 1 shows the conceptual framework as coding tree, and online resource 3 shows the conceptual framework complemented with results and quotes. In this result section we will only elaborate on the most prominent subthemes per main theme; minor subthemes can be consulted in online resource 3.

#### Before initial ED visit

This theme included several subthemes, of which health status and social system were most prominent. The patients expressed variation in the experienced health status, from “already feeling very sick” before their initial ED-visit to feeling”in a good physical condition”. A second subtheme was the social environment of the patients. The majority felt they had a solid social system around them, which is illustrated by the following quote: “*Well, my social network is just like that. Most of them are older and yes, everyone takes care of each other. If someone cannot go to the store, than I will do that for her.”*(interview 1, hereafter: I1)*.*

#### Initial ED visit

Waiting times and communication were the most important subthemes within this main theme. Waiting time was a major subject in all the interviews. Many patients had negative experiences: *“Oh no, it is not emergency, because you have to wait regardless. You have to wait, wait, wait, but it takes too long.”*(I3). Some patients add to this that the waiting times are particularly bothersome, as they were not informed what they were waiting for: *“The unnecessarily long waits, I was there all night. Look, if you do not know why, it feels like useless waiting.”*(I1), and *“But if someone would just explain that it is busy and why, then you know you will have to wait. No, there was not a lot of communication”* (I3)*.*

The ‘communication between the patient and healthcare professionals’ was highly dependent not only on the attending healthcare professionals during the visits, the demand on the ED, but also on the preferences and sickness level of the patient. An example of a positive experience in which the patient was reassured is as follows: *“Antibiotics, I told them not to give me those. I am extremely afraid of it. But then the physician came in, she told me that I really did not have to be afraid, that they checked it really well. She explained that the medicine that gives me the allergic reaction is not in it.”* (I7). However, other patients had a more negative experience regarding the communication with healthcare professionals, in particular the patient in interview 1: *“No, no questions were asked. There was no interest whatsoever. They do not listen”.*

#### Between ED visits

The most apparent subthemes here were aftercare and general practitioner (GP). Aftercare after the initial ED visit included experiences regarding a variety of aftercare activities (e.g. outpatient appointments, home care, etc.). Some of the patients had negative experiences with the aftercare. For example, the patient in interview 6, of whom the healthcare staff already doubted his ability to go home independently after his initial visit. He was eventually discharged home without aftercare, resulting in a negative experience as he proved insufficiently independent. *“After the second visit it was very clear that I was not able to function at home independently, so the staff contacted the transfer ward.”*.

The contact with the GP during this phase was related to the previous experience before the initial ED-visit. Some patients expected their GP to take on an overall monitoring role in their healthcare journey, which did not always happen: “*As far as I know, the GP is supposed to take over some things from the hospital and he should be more attentive to the patients. That is the new standard. But in this case I have not experienced this with him, until he called last week to ask what had happened.”*(I12)*.* On the other hand, some patients felt GP care is unnecessary when being treated in the hospital: *“I think it is not logical if the GP gets involved, if the hospital is already taking care of your treatment.”* (I1)*.*

#### Return ED visit

For the ‘return ED visit’, waiting time and discharge were the most important subthemes. Overall, the waiting times were still experienced as long and bothersome during the return visit by most patients.

While after the initial ED visit the majority of patients was discharged home, after the return visit the majority of patients was admitted to the hospital. During the interview, patients were asked whether they were comfortable with being discharged (from either the ED or ward). In some cases, patients felt that the discharge from the initial visit was ‘too soon’ or that ‘something went wrong during discharge’. This could have had multiple reasons, for example one patient who was supposed to be admitted to the ward after the initial ED visit, but there were no available beds. The physician then decided that the necessity of an admission was not urgent enough to look further. Additionally, sometimes the discharge instructions (i.e. prescription and/or instructions for medication) was suboptimal: *“So then I took the wrong dose for three days and the pain persisted. I called and was then asked how many tablets I took per day. She told me I was supposed to take three a day, but the box said one.”* (I10)*.*

#### After return ED visit

Social system and expectations were the most apparent subthemes in this main theme.

In some patients the deteriorated health condition also impacted other aspects of their life, like for example the ‘social system’: *“For the time being we do not want any visitors, other than family and the kids. That is too much for now.”* (I2). Other patients realize that with ageing comes the loss of social contacts: *“Everyone around me disappeared. Most of them died, and now many young people live here, who go to work. They do not have time, they never have time.”* (I11).

The subtheme ‘expectations’ encompassed the process of coping and/or acceptance with the corresponding (health) outcomes after two ED visits. These expectations included the impact on their life in general, quality of life after the ED, and worries regarding these concepts. Examples are as follows: *“(The ED visits) really had an impact on me. I had hoped for more: that I could walk and do more. Now it is all so restricted.”* (I7). *“On the inside it is very hard for me, and I am scared. How will it all go? What will happen? How much pain will I be in?”* (I8).

#### Overarching experiences at ED

Besides experiences related to specific situations in their journey, we also asked patients about their overarching experiences at the ED and improvement initiatives.

Examples of a ‘positive overarching experience’ are as follows: *“Well, I am obviously upset when I have to go (to the ED), but I do not feel like that once I am there. It is relaxed, I would say you feel safe.”* (I11).

The same goes for overarching ‘negative experiences’ at the ED, for example: *“The staff is hidden from the patients, they are far away, around the corner. The old ED, (before renovation) had a very nice ambiance. Now you feel lonely, you only get to see people passing by every now and then.”* (I12)*.*

All patients were asked about potential ‘improvement initiatives’ that could increase patient satisfaction in the future. Many of the improvement initiatives mentioned were regarding the waiting times as follows: *“Simply explain to patients how long they will have to wait, and maybe come up with a reason why the waiting times are so long.”*(I1). Others were regarding discharge as follows: “*Often you go home (from the ED) while there is unfinished business. And most of the times when you actually are getting admitted, you still go home with unfinished business while there actually is something wrong. I would say just let a doctor come, let him examine you while you are at home*” (I5)*.* Ambiance and approach by the healthcare professionals were also mentioned as follows: *“They have to be a little more spontaneous and open. It all has to be more human-centered, friendly, it feels so formal now”*(I1).

**Fig. 2:**
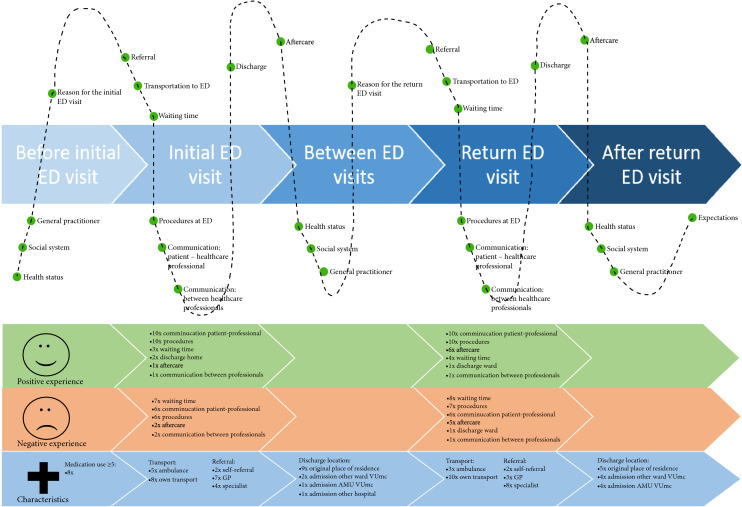
Visual representation of the patient journey of older people with a return ED visit within 30 days. *The upper part of the figure is a visual representation of the trajectory. The lower part incorporated the positive and negative experiences and medical characteristics per phase, in which the numbers represent the number of patients who voiced their experience regarding a subtheme. The numbers do not add up to 13 as one patient could mention multiple subthemes

Figure 1 visualizes the journey of all 13 patients in a patient journey map that shows experiences, both positive and negative, and characteristics for each phase (Fig. 2).

## Discussion

This study provides a chronological overview of the patient journey of older patients from the initial to the return ED visit within 30 days. It provides insight into positive and negative experiences and perspectives of these patients regarding their journey. We derived multiple main-, supplementary-, and sub-themes from our data and developed a conceptual framework with a chronological timeline as basis, in accordance with the patient journey (online resource 3). This study has shed light on some issues that older patients experience with multiple facets of the ED system. Focusing on the improvement of these issues might contribute to an enhanced patient experience and thereby contribute to patient-centered care. The two most apparent issues were the waiting time and discharge communication.

The waiting time was mentioned for both the initial and return visit, and by every interviewed patient and by every interviewed patient, sometimes positively, sometimes neutral, but mostly negatively. This is in line with many other studies on patient experiences at the ED as described in, i.e. literature reviews [37, 38]. Our qualitative patient journey approach adds to this predominantly quantitative ED literature, as we focused on *how* they experienced the visit (i.e. the waiting times), and *why* it was experienced this way. The experiences regarding waiting time in our study were similar for both visits. Literature shows that the absolute waiting times (minutes wait) are highly dependent on the demand on the ED, the attending staff, and the triage code (i.e. urgency). For the perceived waiting times the patient’s former ED experiences and expectations are important factors [39]. Studies have shown that perceived waiting times and not being informed about the waiting times have a larger impact on patient experience and satisfaction than objective waiting times [40, 41]. This indicates that clear communication regarding waiting times is important and to make sure that patients do not feel like they are forgotten and excluded. It is notable that in our study it was often unclear for patients what or whom they were waiting for. This potentially contributed more to having a negative experience with waiting time, than the actual minutes wait.

For almost all interviewed patients the reason for the return visit was related to the reason for the initial visit, as judged by the patients themselves. Some of these patients were faced with negative experiences because of insufficient discharge instructions and/or aftercare. These findings potentially suggest that some patients might have not received optimal ED care during the initial visit and/or the period between visits. Previous studies show that 30% to 40% of ED return visits could be prevented with appropriate and adequate discharge instructions and aftercare [17, 42, 43].

Based on the results of our study we recommend to provide older patients at the ED with clear communication regarding waiting times. As discussed earlier, waiting times are an important determinant of patient experience and satisfaction, and timely provision of information about the waiting times to the patients can help [26]. A potential solution to achieve this may be implementing a time-out at the ED every two hours, where healthcare professionals can discuss the status of patients at the ED at that moment and inform the patients regarding the progress. Studies have shown that time-based interventions at the ED have multiple benefits, e.g. helping healthcare professionals to have a clear overview of the ED and workload [44, 45]. Moreover, we believe that such an intervention would provide patients with clarity regarding the course and their health condition, while also ensuring they feel seen and heard. This could potentially lead to enhanced patient experience and contribute to patient-centered care [26].

Furthermore, we recommend to focus on providing clear discharge communication at the ED that is patient-centered, i.e. includes topics that matter and are helpful to patients. Studies have shown that clear discharge communication can lead to efficient continuity of care, a smooth transition from hospital to home, and a reduction of return visits and readmissions [42]. It could also help increase the trust in healthcare professionals and the care received, as research shows that lack of communication can lead to distrust from patients [46]. Moreover, it could help to involve the older patients in their care and transition from hospital to home (i.e. by giving the patients a say in their aftercare etc.), which has also been shown to be effective in preventing readmissions and making the patient feel included [47]. Verbal discharge instructions might not always be sufficient, as verbal-only information is often not recalled correctly by the patient [48]. For these reasons, we recommend a written checklist for checkout when older patients are discharged from the ED. Such a checklist should be comprehensive and clear, by including the information needed and wanted by the patient in understandable and simple language [48, 49]. The ED is a crowded and hectic environment, which can make it challenging to implement interventions that require time from healthcare professionals who are already dealing with high workload. However, a checklist for checkout, though comprehensive, does not necessarily have to be long and/or time-consuming. We believe that if three main topics (diagnosis or diagnostic uncertainty; treatment plan; follow-up instructions) are included, this could be feasible for healthcare professionals to conduct, whilst still having potential for positive effects for patients. Moreover, as is the case with any intervention, it is important that there is a suiting implementation strategy in place, integrating both (implementation) evidence and end-user (healthcare professionals and patients) perspectives. The use of the Consolidated Framework For Implementation Research could be of use for this [50]. Discharge communication by the means of a checklist for checkout can be amplified by post-discharge tracking, for example by telephone follow-up. Though RCTs by Biese et al. show no effect of telephone follow-up on health outcomes [51, 52], other studies suggest that telephone follow-up can provide older patients with social and emotional support and increase patient satisfaction [53–55]. We, therefore, believe that telephone follow-up has potential in contributing to a positive experience for older patients.

It could be argued that the aforementioned quality improvements might be particularly important and helpful for frail older patients that present at EDs, as frail older patients are at higher risk of adverse outcomes than non-frail older patients [56]. Moreover, frailer older patients endure more negative experiences, especially in the transition from the hospital to home [57]. Therefore, it could be argued that these interventions should focus on frail older patients first. However, since there is no internationally accepted gold standard for identifying frailty in older patients, and studies report a varying prevalence of frailty at the ED, ranging from 7 to 80% [58], we believe quality improvements should be generically implemented for all older patients that present at EDs.

A strong aspect of this study is the use of the patient journey mapping method, allowing us to measure and visualize the patient’s experiences and perspective during their journey from before the initial ED visit until the return ED visit. This provided us with the opportunity to identify issues, without losing chronology, detail, complexity, and context. It adds to the predominantly quantitative literature by adding in-depth data and results regarding the complex phenomenon in ED care from the patient perspective. Another strength of our study is that all data were coded independently by two coders, followed by a comprehensive consensus procedure, therefore maximizing reliability and credibility of our results. Moreover, the coding team consisted of one medical doctor (BD) and one psychologist (BS) to account for differences in interpretation and to capture both the medical and psychological components. Additionally, the majority of the interviews were conducted in the patient’s home environment, supporting patients to feel comfortable and, therefore, potentially resulting in more productive interviews.

Despite the strengths of this study, there are some limitations to take into consideration. First, interviews that require looking back on a period in the past could be subject to multiple sources of bias. For example, our data could contain hindsight bias, as the patients knew their outcomes of both the initial and return visit, which could have influenced the memory of the initial experiences. Second, especially as our participants were all older patients, there might have been some issues with cognition and memory. However, patients were mostly interviewed within one week (but never longer than two weeks) after the return ED visit, and patients with apparent cognitive impairment were excluded from participation, in order to minimize the chance of recall bias.

Future research should focus on testing multiple potential interventions (i.e. time-out, checklist for checkout, post-discharge tracking) in order to determine which (combination of) interventions work best for this group of older patients. These studies should seek to not only collect quantitative data, but also enrich it with qualitative data and patient-centered outcomes to include the perspectives of the patients and healthcare professionals. We suggest to perform patient journey studies again, to determine whether these interventions really contribute to a more positive experience and patient-centered care.

## Conclusion

This study aimed to provide insight into the experiences and perspectives of older patients from their initial to their return ED visit (within 30 days) by patient journey mapping. The two major findings were that lack of information about waiting times and suboptimal discharge communication contributed to negative experiences. These findings are in line with previous, mainly quantitative, ED studies, but the qualitative patient journey approach enabled us to look at these complex phenomenon in detail and provides the reasons behind the experiences. Moreover, the patient journey method allowed us to shed light on these issues from a patient perspective and to pinpoint solutions that could contribute to a positive experience and enhanced patient-centered care in the future. These potential solutions include the following: a time-out at the ED every two hours and clear and comprehensive discharge communication.

## Supplementary Information

Below is the link to the electronic supplementary material.

Supplementary file1 (DOCX 32 KB)

## Data Availability

Data are available upon reasonable request after approval of the corresponding author.

## Declarations

The authors state that the methods of this study were carried out in accordance with relevant guidelines and regulations. The COREQ checklist for qualitative research was used.

## References

[1] (2013) Policy statement. Ann Emerg Med 61(6):726–727. 10.1016/j.annemergmed.2013.03.037. ISSN 0196-0644 10.1016/j.annemergmed.2013.03.037 23684339

[2] Morley C, Unwin M, Peterson GM, Stankovich J, Kinsman L (2018). Emergency department crowding: a systematic review of causes, consequences and solutions. PloS One.

[3] Pines JM, Griffey RT (2015). What we have learned from a decade of ED crowding research. Acad Emerg Med Off J Soc Acad Emerg Med.

[4] NZa (2019) Monitor acute zorg 2018. Nederlandse Zorgautoriteit 2019

[5] Legramante JM, Morciano L, Lucaroni F, Gilardi F, Caredda E, Pesaresi A (2016). Frequent use of emergency departments by the elderly population when continuing care is not well established. PloS One.

[6] Kaskie B, Obrizan M, Jones MP, Bentler S, Weigel P, Hockenberry J (2011). Older adults who persistently present to the emergency department with severe, non-severe, and indeterminate episode patterns. BMC Geriatr.

[7] Niska R, Bhuiya F, Xu J (2010). National hospital ambulatory medical care survey: 2007 emergency department summary. Natl Health Stat Rep.

[8] Aminzadeh F, Dalziel WB (2002). Older adults in the emergency department: a systematic review of patterns of use, adverse outcomes, and effectiveness of interventions. Ann Emerg Med.

[9] Karam G, Radden Z, Berall LE, Cheng C, Gruneir A (2015). Efficacy of emergency department-based interventions designed to reduce repeat visits and other adverse outcomes for older patients after discharge: a systematic review. Geriatr Gerontol Int.

[10] McCusker J, Karp I, Cardin S, Durand P, Morin J (2003). Determinants of emergency department visits by older adults: a systematic review. Acad Emerg Med Off J Soc Acad Emerg Med.

[11] Gray LC, Peel NM, Costa AP, Burkett E, Dey AB, Jonsson PV (2013). Profiles of older patients in the emergency department: findings from the interRAI multinational emergency department study. Ann Emerg Med.

[12] Caplan GA, Brown A, Croker WD, Doolan J (1998). Risk of admission within 4 weeks of discharge of elderly patients from the emergency department—the DEED study. Discharge of elderly from emergency department. Age Ageing.

[13] Lowthian J, Straney LD, Brand CA, Barker AL, Smit Pde V, Newnham H (2016). Unplanned early return to the emergency department by older patients: the Safe elderly emergency department discharge (SEED) project. Age Ageing.

[14] de Gelder J, Lucke JA, de Groot B, Fogteloo AJ, Anten S, Heringhaus C (2018). Predictors and outcomes of revisits in older adults discharged from the emergency department. J Am Geriatr Soc.

[15] Duseja R, Bardach NS, Lin GA, Yazdany J, Dean ML, Clay TH (2015). Revisit rates and associated costs after an emergency department encounter: a multistate analysis. Ann Intern Med.

[16] Probst MA, Kanzaria HK, Schoenfeld EM, Menchine MD, Breslin M, Walsh C (2017). Shared decisionmaking in the emergency department: a guiding framework for clinicians. Ann Emerg Med.

[17] Han CY, Lin CC, Goopy S, Hsiao YC, Barnard A (2017). Elders’ experiences during return visits to the emergency department: a phenomenographic Study in Taiwan. Nurs Res.

[18] Fluitman KS, van Galen LS, Merten H, Rombach SM, Brabrand M, Cooksley T (2016). Exploring the preventable causes of unplanned readmissions using root cause analysis: coordination of care is the weakest link. Eur J Intern Med.

[19] Cleary PD (2016). Evolving concepts of patient-centered care and the assessment of patient care experiences: optimism and opposition. J Health Polit Policy Law.

[20] Institute of Medicine Committee on Quality of Health Care in A (2001) Crossing the Quality Chasm: A New Health System for the 21st Century. Washington (DC): National Academies Press (US) Copyright 2001 by the National Academy of Sciences. All rights reserved

[21] Bertakis KD, Azari R (2011). Patient-centered care is associated with decreased health care utilization. J Am Board Fam Med: JABFM.

[22] Gluyas H (2015). Patient-centred care: improving healthcare outcomes. Nurs Stand (Royal Coll Nurs (Great Britain):1987).

[23] Stone S (2008). A retrospective evaluation of the impact of the Planetree patient-centered model of care on inpatient quality outcomes. HERD.

[24] Hansson E, Ekman I, Swedberg K, Wolf A, Dudas K, Ehlers L (2016). Person-centred care for patients with chronic heart failure—a cost-utility analysis. Eur J Cardiovasc Nurs: J Work Group Cardiovasc Nurs Eur Soc Cardiol.

[25] Sonis JD, White BA (2020). Optimizing patient experience in the emergency department. Emerg Med Clin North Am.

[26] Graham B, Endacott R, Smith JE, Latour JM (2019). ‘They do not care how much you know until they know how much you care’: a qualitative meta-synthesis of patient experience in the emergency department. Emerg Med J: EMJ.

[27] Trebble TM, Hansi N, Hydes T, Smith MA, Baker M (2010). Process mapping the patient journey: an introduction. BMJ (Clin Res ed).

[28] Driesen B, Merten H, Wagner C, Bonjer HJ, Nanayakkara PWB (2020). Unplanned return presentations of older patients to the emergency department: a root cause analysis. BMC Geriatr.

[29] Driesen B, Merten H, Barendregt R, Bonjer HJ, Wagner C, Nanayakkara PWB (2021) Root causes and preventability of emergency department presentations of older patients: a prospective observational study. BMJ Open [Internet. 11(8):e049543. Available from: http://europepmc.org/abstract/MED/34348952, 10.1136/bmjopen-2021-049543, https://europepmc.org/articles/PMC8340285, https://europepmc.org/articles/PMC8340285?pdf=render 10.1136/bmjopen-2021-049543 PMC8340285 34348952

[30] Considine J, Smith R, Hill K, Weiland T, Gannon J, Behm C (2010). Older peoples’ experience of accessing emergency care. Australas Emerg Nurs J.

[31] Gordon J, Sheppard LA, Anaf S (2010). The patient experience in the emergency department: a systematic synthesis of qualitative research. Int Emerg Nurs.

[32] Olthuis G, Prins C, Smits MJ, van de Pas H, Bierens J, Baart A (2014). Matters of concern: a qualitative study of emergency care from the perspective of patients. Ann Emerg Med.

[33] Shankar KN, Bhatia BK, Schuur JD (2014). Toward patient-centered care: a systematic review of older adults' views of quality emergency care. Ann Emerg Med.

[34] Nikki L, Lepisto S, Paavilainen E (2012). Experiences of family members of elderly patients in the emergency department: a qualitative study. Int Emerg Nurs.

[35] Robinson C, Verrall C, Houghton L, Zeitz K (2015). Understanding the patient journey to the emergency department—a South Australian study. Australasian Emerg Nurs j: AENJ.

[36] Elo S, Kyngäs H (2008). The qualitative content analysis process. J Adv Nurs.

[37] Sonis JD, Aaronson EL, Lee RY, Philpotts LL, White BA (2018). Emergency department patient experience: a systematic review of the literature. J Patient exp.

[38] Boudreaux ED, O'Hea EL (2004). Patient satisfaction in the emergency department: a review of the literature and implications for practice. J Emerg Med.

[39] Thompson DA, Yarnold PR, Williams DR, Adams SL (1996). Effects of actual waiting time, perceived waiting time, information delivery, and expressive quality on patient satisfaction in the emergency department. Ann Emerg Med.

[40] Sun BC, Adams J, Orav EJ, Rucker DW, Brennan TA, Burstin HR (2000). Determinants of patient satisfaction and willingness to return with emergency care. Ann Emerg Med.

[41] Hedges JR, Trout A, Magnusson AR (2002). Satisfied patients exiting the emergency department (SPEED) study. Acad Emerg Med Off J Soc Acad Emerg Med.

[42] Han CY, Chen LC, Barnard A, Lin CC, Hsiao YC, Liu HE (2015). Early revisit to the emergency department: an integrative review. J Emerg Nurs.

[43] Lerman B, Kobernick MS (1987). Return visits to the emergency department. J Emerg Med.

[44] Burke JA, Greenslade J, Chabrowska J, Greenslade K, Jones S, Montana J (2017). Two hour evaluation and referral model for shorter turnaround times in the emergency department. Emerg med Australasia: EMA.

[45] Morais Oliveira M, Marti C, Ramlawi M, Sarasin FP, Grosgurin O, Poletti PA (2018). Impact of a patient-flow physician coordinator on waiting times and length of stay in an emergency department: a before-after cohort study. PloS One.

[46] Schultz H, Qvist N, Mogensen CB, Pedersen BD (2014). Perspectives of patients with acute abdominal pain in an emergency department observation unit and a surgical assessment unit: a prospective comparative study. J Clin Nurs.

[47] Murray J, Hardicre N, Birks Y, O'Hara J, Lawton R (2019). How older people enact care involvement during transition from hospital to home: a systematic review and model. Health Expect Int J Public Particip Health Care Health Policy.

[48] Hoek AE, Anker SCP, van Beeck EF, Burdorf A, Rood PPM, Haagsma JA (2020). Patient discharge instructions in the emergency department and their effects on comprehension and recall of discharge instructions: a systematic review and meta-analysis. Ann Emerg Med.

[49] Taylor DM, Cameron PA (2000). Discharge instructions for emergency department patients: what should we provide?. J Accid Emerg Med.

[50] Damschroder LJ, Aron DC, Keith RE, Kirsh SR, Alexander JA, Lowery JC (2009). Fostering implementation of health services research findings into practice: a consolidated framework for advancing implementation science. Implement Sci: IS.

[51] Biese K, Lamantia M, Shofer F, McCall B, Roberts E, Stearns SC (2014). A randomized trial exploring the effect of a telephone call follow-up on care plan compliance among older adults discharged home from the emergency department. Acad Emerg Med Off J Soc Acad Emerg Med.

[52] Biese KJ, Busby-Whitehead J, Cai J, Stearns SC, Roberts E, Mihas P (2018). Telephone Follow-Up for Older Adults Discharged to Home from the Emergency Department: A Pragmatic Randomized Controlled Trial. J Am Geriatr Soc.

[53] Cochran VY, Blair B, Wissinger L, Nuss TD (2012). Lessons learned from implementation of postdischarge telephone calls at Baylor Health Care System. J Nurs Adm.

[54] Poncia HD, Ryan J, Carver M (2000). Next day telephone follow up of the elderly: a needs assessment and critical incident monitoring tool for the accident and emergency department. J Accid Emerg Med.

[55] Hwang U, Hastings SN, Ramos K (2018). Improving emergency department discharge care with telephone follow-Up. Does it connect?. J Am Geriatr Soc.

[56] Lahousse L, Maes B, Ziere G, Loth DW, Verlinden VJ, Zillikens MC (2014). Adverse outcomes of frailty in the elderly: the Rotterdam Study. Eur J Epidemiol.

[57] Andreasen J, Lund H, Aadahl M, Sørensen EE (2015). The experience of daily life of acutely admitted frail elderly patients one week after discharge from the hospital. Int J Qual Stud Health Well-Being.

[58] Theou O, Campbell S, Malone ML, Rockwood K (2018). Older Adults in the Emergency Department with Frailty. Clin Geriatr Med.

[59] de Gelder J, Lucke JA, Blomaard LC, Booijen AM, Fogteloo AJ, Anten S (2018). Optimization of the APOP screener to predict functional decline or mortality in older emergency department patients: Cross-validation in four prospective cohorts. Exp Gerontol.

