# Supplementary material for: Experiences and perspectives of older patients with a return visit to the emergency department within 30 days: patient journey mapping
Source: Eur Geriatr Med. 2021 Nov 10;13(2):339–50. doi: 10.1007/s41999-021-00581-6 (PMC9018642; doi:10.1007/s41999-021-00581-6)
Supplement: Supplementary file 1 — Supplementary file1 (DOCX 32 KB) [file 41999_2021_581_MOESM1_ESM.docx]

Article: Experiences of older patients with a return visit to the emergency department within 30 days: Patient journey mapping

Journal: European Journal of Ageing

Authors: Bo Schouten*^1^* ^a^, Babiche E.J.M. Driesen^2 a^, Hanneke Merten*^1^*, Brigitte H.C.M. Burger^3^, Mariëlle G. Hartjes^3^, Prabath W.B. Nanayakkara*^3^*, Cordula Wagner*^14^*

*^1^*Amsterdam UMC, Vrije Universiteit Amsterdam, Department of Public and Occupational Health, Amsterdam Public Health research institute, De Boelelaan 1117, Amsterdam, The Netherlands

*^2^*Amsterdam UMC, Vrije Universiteit Amsterdam, Department of Emergency Medicine, Amsterdam Public Health research institute, De Boelelaan 1117, Amsterdam, The Netherlands

*^3^*Amsterdam UMC, Vrije Universiteit Amsterdam, Section General and Acute Internal Medicine,

Department of Internal Medicine, Amsterdam Public Health research institute, De Boelelaan 1117, Amsterdam, The Netherlands

^4^ Netherlands Institute for Health Services Research (NIVEL), Utrecht, The Netherlands

^a^ both authors contributed equally to this work

Corresponding author: Bo Schouten, [b.schouten1@amsterdamumc.nl](mailto:b.schouten1@amsterdamumc.nl) , +3120-4441737, P/O box 7057, 1007 MB Amsterdam, The Netherlands, ORCID: 0000-0001-6387-7633

**Online resource 1.** Topic list

| **Topic** | **Questions** |
| --- | --- |
| **Introduction** | - Introduction by interviewer; - Explanation of purpose of the interview and study; - Providing information regarding the transcription process and anonymization of the data; - Providing an estimate of the duration of the interview. |
| **Get acquainted with the patient** | - Sex/age/marital status? - What is your living situation?   - Do you receive household support?   - Do you receive nursing homecare? |
| **Support system** | - Could you tell something about your social network?   - How many times a day/week are you in contact with other people? And if so, with whom, and what kind of contact do you have with them?     - (family/friends/neighbors/ volunteers/ hobby club/ sport/ patient organization)?     - (by phone; physical contact; electronic contact)?   - Could you tell something about your contact with your general practitioner? (date last contact/ your relation) |
| **The time prior to the first visit to the emergency department** | - How did you experience your health status (sickness experience)? - Who did you speak to in the 24 hours period prior to the visit - What was your experience of access to the emergency department? (use; reluctance; resistance; transfer) - Were there barriers/ was there a lack of something during this period? |
| **The time during the first visit to the emergency department** | Arrival:   - What was the reason of your visit to the emergency department? - How did you arrive at the emergency department (referral method/ which transport/ with guidance/ urgency)? - What was your experience at the arrival of the emergency department? (Did you feel welcome?)   Visit at the emergency department   - Could you tell something about your visit at the emergency department? - Timetable   - How long did you spend at the emergency department?   - How long was the waiting time at the emergency department (time from arrival to triage; from triage to see a nurse and doctor; from seeing a doctor to get an examination and treatment plan; to discharge to ward or home) - Communication   - In what way was the information obtained during this visit? (Was everything clear?)   - Could you tell something about the communication between you and the healthcare workers? (nurse/doctor; become involved in the care process; the opportunity to tell your story and enter into a dialogue; respect; staff listen carefully; your opinion)   - What was your participation during your first visit to the emergency department (were you involved as much as you wanted in your counselling or treatment?)   Discharge   - How did you experience the discharge process?   (Did you agree with the date you were discharged from the hospital? What kind of treatment did you receive at discharge to home? Did you understand the treatment (medicine or other aftercare), the diagnosis, when you must came back, the expected course / questions were (un)answered)   - Was the advice feasible for you? Did you ran into problems during your treatment at home? - Was the care sufficient at the emergency department? What made it (not) sufficient/adequate? Did you feel comfortable? (spend enough time) |
| **The time between the first and the second visit to the emergency department** | - Could you tell something about the time between your first and second visit at the emergency department?   - How many days did this period last?   - What consequences did your first visit to the emergency room have for your daily life/ your family/ …?   Has there been a change in the number of contacts after your first visit to the emergency department? (who, when, what kind of contact)   - - How did you feel when you were back home?   - How was the follow-up organized?   - Were you able to apply the treatment and discharge instructions after the first visit?   - How did you experience your mental health status (Sickness experience)? - What was your experience of access to the emergency department? (use; reluctance; resistance; transfer) |
| **The time during the second visit to the emergency department** | Arrival   - What was the reason of your visit to the emergency department? - How did you arrive at the emergency department (referral method/ which transport/ with guidance/ urgency)? - What was your experience at the arrival of the emergency department? (Did you feel welcome?)   Visit at the emergency department   - Could you tell something about your visit at the emergency department? - Timetable   - How long did you spend at the emergency department?   - How long was the waiting time at the emergency department (time from arrival to triage; from triage to see a nurse and doctor; from seeing a doctor to get an examination and treatment; to discharge to ward or)). - Communication   - Could you tell something about the communication between you and the healthcare workers? (nurse/doctor; become involved in the care process; the opportunity to tell your story and enter into a dialogue; respect; staff listen carefully; your opinion)   - In what way was the information obtained during this visit? (Was everything clear?)   - What was your participation during your first visit to the emergency department (were you involved as much as you wanted in your counselling or treatment?)   Discharge   - How did you experience the discharge process?   (Did you agree with the date you were discharged from the hospital? What kind of treatment did you receive at discharge to home? Did you understand the treatment (medicine or other aftercare), the diagnosis, when you must came back, the expected course / questions were (un)answered)   - Was the advice feasible for you? Did you ran into problems during your treatment at home? - Was the care sufficient at the emergency department? What makes is (not) sufficient/adequate? Did you feel comfortable? (spend enough time) |
| **The time after the second visit to the emergency department** | - Could you tell something about the time after your second visit at the emergency department?   - How did you experience your mental health status (Sickness experience)?   - Has there been a change in the number of contacts after your first visit to the emergency department? (who, when, what kind of contact) |
|  | - How did you experience the overall flow beginning from the day before your first visit until two days after your second visit within 30 days at the emergency department? - What do you think is the most important subject at the emergency department for a good experience for older patients? - In general, how would you rate your overall mental health status now? - Do you have ideas how to improve the care of older patients at the emergency department? |

**Online resource 2.** Patient and emergency department visits characteristics

| **Patient characteristics** | **n=13** | |
| --- | --- | --- |
| **Age in years, median (IQR)** | 79 (7) | |
| 70-79, n (%) | 7 (53.8%) | |
| 80-89, n (%) | 5 (38.5%) | |
| 90+, n (%) | 1 (7.7%) | |
| **Male, n (%)** | 8 (61.5%) | |
| **Living with partner, n (%)^a^** | 8 (61.5%) | |
| **Home care, n (%)^a^** | 3 (23.1%) | |
| **Medication use ≥5, n (%)^a^** | 8 (61.5%) | |
| **GP visits in the previous year, median (IQR)^a^** | 2 (3) | |
| **ED visits in the previous year, median (IQR)^a^** | 2 (2) | |
| **Patients with a hospital admission^a^** |  | |
| < 30 days, n (%) | 9 (69.2%) | |
| < 6 months, n (%) | 13 (100%) | |
| < year, n (%) | 13 (100%) | |
| **Specialist treatments in the previous year^a^ , median (IQR)** | 2 (9) | |
| **Deceased within the 6 months follow-up period, n (%)^a^** | 2 (15.4%) | |
|  |  | |
| **ED visits characteristics** | **Initial visit** | **Return visit** |
| **Referral** |  |  |
| Self-referral, n (%) | 2 (15.4%) | 2 (15.4%) |
| GP, n (%) | 7 (53.8%) | 3 (23.1%) |
| Specialist, n (%) | 4 (30.8%) | 8 (61.5%) |
| **Transport** |  |  |
| Ambulance, n (%) | 5 (38.5%) | 3 (23.1%) |
| Own transport, n (%) | 8 (61.5%) | 10 (76.9%) |
| **Triage code, to be seen:** |  |  |
| Directly, n (%) | 1 (7.7%) | 0 (0%) |
| Within 10 minutes, n (%) | 6 (46.1%) | 7 (53.8%) |
| Within 1 hour, n (%) | 3 (23.1%) | 4 (30.8%) |
| More than 1 hour, n (%) | 3 (23.1%) | 2 (15.4%) |
| **Triage complaint** |  |  |
| Abdominal pain, n (%) | 1 (7.7%) | 1 (7.7%) |
| Thorax pain, n (%) | 2 (15.4%) | 1 (7.7%) |
| General pain, n (%) | 3 (23.1%) | 2 (15.4%) |
| Dyspnea, n (%) | 1 (7.7%) | 1 (7.7%) |
| Hematuria, n (%) | 1 (7.7%) | 1 (7.7%) |
| Fever, n (%) | 3 (23.1%) | 3 (23.1%) |
| Collapse, n (%) | 1 (7.7%) | 1 (7.7%) |
| Luxation hip, n (%) | 1 (7.7%) | 1 (7.7%) |
| Wound, n (%) | 0 (0%) | 1 (7.7%) |
| Cardiac dysrhythmia, n (%) | 1 (7.7%) | 1 (7.7%) |
| **Diagnostic tests at ED, median (IQR)** | 2 (3) | 2 (2) |
| **Consultations at ED, median (IQR)** | 2 (2) | 1 (1) |
| **APOP decline score %, median (IQR)** |  | 25 (21) |
| Low: <60%, n (%) | Not available | 11 (85%) |
| High: >60%, n (%) | Not available | 2 (15.4%) |
| **APOP mortality score %, median (IQR)** |  | 7.5 (10) |
| Low: <60%, n (%) | Not available | 13 (100%) |
| High >60%, n (%) | Not available | 0 (0%) |
| **Discharge from ED** |  |  |
| Original place of residence | 9 (69.2%) | 5 (38.4%) |
| Admission acute medical unit VUmc | 1 (7.7%) | 4 (30.8%) |
| Admission other ward VUmc | 2 (15.4%) | 4 (30.8%) |
| Admission other hospital | 1 (7.7%) | 0 (0%) |
| **Days between initial and return visit, median (IQR)** | 9 (17) | |
| ≤2 days | 3 (23.1%) | |
| 3-7 days | 4 (30.8%) | |
| >7 days | 6 (46.1%) | |
|  |  | |

IQR = Inter Quartile Range, ED = emergency department, GP = general practitioner

^a^ = return visit as target

**Online resource 3.** Conceptual framework

| MAIN THEME | SUBTHEME | RESULTS | QUOTES |
| --- | --- | --- | --- |
| Before emergency department (ED) visit | Health status | The participations expressed variation in the experienced health status, from already feeling very sick before their initial ED visit to feeling in a good physical condition. | *“I might have passed 80, but I am differently 80 than another 80 year old.”* (I 1)  *“I do not feel like an elderly person.”* (I 13) |
|  | Social system | The majority felt they had a solid social system around them. | “*Well, my social network is just like that. Most of them are older and yes, everyone takes care of each other. If someone cannot go to the store, than I will do that for her. That is how it goes here.”* (I 1)  *“Yes, we have people coming over regularly.”* (I 10) |
|  | General practitioner (GP) | The contact with the GP varied between patients. Some participants indicated they had little to no contact with their GP before the initial ED visit, whereas others had frequent contact with their GP. | *“No that is a miss. He saw me when I got home after surgery, when he came in to check, but after that I have not heard from him up until last week.”* (I 12)  *“(the contact with the GP) is very good, very good indeed. I have a great GP. He is a very good man. That is definitely worth something.”* (I 9) |
|  | Reason for the initial ED visit | The complaints that led to the ED visit, these were different for every patient. | *“And then [nephrologist] saw me coming in, he was shocked by my face, pale, he said I could not go home like this. He said my kidney was functioning fine, but that I could not proceed like this. Then I got picked up and went to the ED.”* (I 8) |
| Initial ED visit | Referral | There were three options of referral: GP, medical specialist or self-referral. Most patients were referred by their GP. | *“I got sent by the GP”* (I 7)  *“So he (nephrologist) called and I had to go to the ED.”* (I 8) |
|  | Transportation to ED | Most patients got to the ED with their own transport. Some patients went by ambulance, none of whom with sirens. | *“Then I was brought by ambulance.”* (I 1) |
|  | Waiting time | The waiting time at the ED was a major subject in all the interviews. Many patients had negative experiences with the waiting time. Some patients add to this that the waiting time was particularly bothersome, as they were not informed what they were waiting for. | *“Every second feels like an hour, especially since you are not feeling well.”* (I 2)  *“The thing is, you wait way too long. Why, I do not know. There are enough physicians I think, but yes, (the physician) was running from left to right, north to south in the hospital. She was the only one there I think.”* (I 8) |
|  | Procedures at ED | This theme is very broad and contains everything related to the procedures during ED visits, for example the flow (from waiting room, triage, to a room at the ED), the diagnostics, and the diagnosing. This was different for every patient. | *“They start by giving you a (identification) band, that way you cannot escape.”* (I 5)  *“You get a band, because they have already passed on the information. Then you stay in the waiting room and wait for your turn.”* (I 4)  *“Every hospital is always very thirsty for blood, because first thing is that they try to draw blood from you.”* (I 5) |
|  | Communication: patient – healthcare professional | The communication between the patient and healthcare professionals was highly dependent on the attending healthcare professionals during the visits, the demand on the ED, but also on the preferences and sickness level of the patient. | *“They do not take you for a fool to whom they would say: well sir we know it all, you get this and do not ask why. No, they have told me what (the treatment) is for and what the consequences could be.”* (I 10)  *“It was more information than I could digest sometimes, than was necessary. Also the wrong information at the end, that was a student, she handled my discharge. I am XX cm tall and weigh XX kg, but the information (in the file) was of another patient.”* (I 12) |
|  | Communication: between healthcare professionals | Communication between healthcare professionals included: communication between specialists, specialist-nurse, between hospitals, and specialist-GP. In this subtheme we also saw much variation in positive/negative experiences between patients. | *“I now was admitted at the VU and they know what it was, because of course (the other hospital) instantly passed on the information. (The other hospital) and VU are connected very well.”* (I 7)  *“The paperwork was not forwarded by (other hospital), and that was very bothersome.”* (I 9) |
|  | Discharge | This included place of discharge (home, admission to the hospital, or to another healthcare facility), discharge instructions, and whether the patient was comfortable with the discharge. In most cases the patients could go home after the initial ED visit. None of the interviewed patients were discharged to another healthcare facility. | *“(The nurse) said madam I do not see a reason to keep you here, you can go home. I said thank you.”* (I 8)*.*  *“Nothing had changed in my condition [during the initial visit]. [I] felt miserable when I went to the ED, while I was there, and still when I went home.”* (I 5)  *“They wanted to admit me. They always admit you to the acute medical unit (AMU) first.”* (I 10)  *“I got discharged home with medication. Colchiline, for gout, with ibuprofen*. *You can take paracetamol with that as long as you do not trespass 4000 mg per 24 hours, so I will not do that.”* (I 10) |
| Between ED visits | Aftercare | This includes experiences regarding a variety of aftercare activities (e.g. outpatient appointments, home care, etc.). In some of the patients, it seemed that a lack of appropriate and/or sufficient aftercare may have contributed to the return visit. | “*The aftercare, I think it is very bad. It is a good thing that I went back [to the ED] myself, because they do not look after you at all. In six weeks you can come back, but I think that is too long. It would have been nice to have an outpatient appointment earlier*.” (I 7)  *“The aftercare needs to get better, because I walked out of the hospital just like that”* (I 7)  *“At first I had my doubt about home care, I had heard stories from a couple of people and thought: ugh. However, these are excellent people, very good.”* (I 12) |
|  | Health status | There was a lot of variation regarding the patients ‘health status’ during this phase. Some patients never stopped feeling sick between ED visits, whereas other patients did not feel sick, indicating that the return visit was less expected. | *“I was already so sick at home, completely sick, the only thing I did was sleeping and turning nauseous.”* (I 8)  *“In that period I had been riding my bicycle and performed all kind of activities, I went grocery shopping by bicycle. Going outside much, nicely in the sun. I did not feel sick.”* (I 13) |
|  | Social system | The majority felt they had a solid social system around them. None of the interviewed patients experienced differences in their social system when comparing the period before, between or after their ED visits. However, some patients indicated that the deteriorated health condition also impacted other aspects of their life. | See before ED visit - social system, and main text for quotes. |
|  | GP | The contact with the GP during this phase was related to the previous experience before the initial ED visit. In addition, some patients expected their GP to take on an overall monitoring role in their healthcare journey, which did not always happen. However, other patients feel GP care is unnecessary when being treated in the hospital. | See before ED visit - GP, and main text for quotes. |
|  | Reason for the return ED visit | The reasons that led to the ED return visit, differed for every patient. Of 13 patients, 12 indicated that the reason for their return visit was related to the reason of the initial visit (i.e. similar complaints, as judges by the patients themselves). | *“the wound was leaking”* and *“One week later the leak was much bigger, and then I went to the ED for the second time.”* (I 13) |
| Return ED visit | Referral | There were three options of referral: GP, medical specialist or self-referral. Most patients were referred by their GP. There were no notable differences compared to the referrals in the initial visit. | See initial ED visit - referral for quotes. |
|  | Transportation to ED | Most patients got to the ED with their own transport. Some patients went by ambulance, none of whom with sirens. There were no notable differences compared to the transportation in the initial visit. | *“We went with our own transport both times.”* (I 5) |
|  | Waiting time | In the return ED visit, the waiting times were still/again experienced as long and bothersome by most patients, and we did not identify an enhanced understanding. | See initial ED visit - waiting, and main text for quotes. |
|  | Procedures at ED | There is a lot of variation between patients, as this is dependent on many factors (i.e. complaint, the communication of the healthcare professional, etc.). When comparing experiences of the initial to those of the return visit, we did not identify notable changes. Some patients remember the procedures from the initial or other prior visits, whereas other patients do not understand why the same procedures have to be repeated in the return visit. | *“And then they performed all those procedures once again, that is such a waste. It is all duplicated.”* (I 2)  *“If I remember it right they drawn blood, took a urine sample, and I also got a scan. While the problem was rather clear to me, I have esophageal cancer, and that is where it comes from.”* (I 6) |
|  | Communication: patient – healthcare professional | The communication between the patient and healthcare professionals was highly dependent on the attending healthcare professionals during the visits, the demand on the ED, but also on the preferences and sickness level of the patient. We did not identify any notable differences between visits, except that some patients were recognized by the healthcare team in the return visit. | *“The ED, they are busy, but they did come in to check on me, to have a chat: “oh you are here again, we know each other” or something like that.”* (I 11) |
|  | Communication: between healthcare professionals | Communication between healthcare professionals included: communication between specialists, specialist-nurse, between hospitals, and specialist-GP. In this subtheme we also saw much variation in positive/negative experiences between patients. There was no notable difference with the experiences in the initial visit, except that in a few (articulate) patients a negative initial experience could trigger them to actively monitor and/or seek a solution in the return ED visit. | *“The second time everything went well. The GP is well informed this time.”* (I 2) |
|  | Discharge | This included place of discharge (home, admission to the hospital, or to another healthcare facility), discharge instructions, and whether the patient was comfortable with the discharge. While after the initial ED visit the majority of patients was discharged home, after the return visit the majority of patients was admitted to the hospital. | *“I got referred to the ward very quickly this time”* (I 5) |
| After return ED visit | Aftercare | This includes experiences regarding a variety of aftercare activities (e.g. outpatient appointments, home care, etc.). There was no notable difference with the experiences in the initial visit. | See between ED visit – aftercare, and main text for quotes. |
|  | Health status | After the two ED visits, some patients felt like they were getting better, whereas other patients realized that the cause for the ED visits (permanently) impacted their health condition. | *“In general I am currently experiencing a repercussion, I am at a 5 as compared to normally. I am back at square one. Physically I am not feeling well, you take a hit, unimaginable.”* (I 12) |
|  | Social system | The majority felt they had a solid social system around them. None of the interviewed patients experienced major differences in their social system when comparing the period before, between or after their ED visits. However, some patients indicated that the deteriorated health condition also impacted other aspects of their life. | See before ED visit - social system, and main text for quotes. |
|  | GP | The contact with the GP varied between patients and was dependent on the contact before and between the ED visits. | See before ED visit - GP, and main text for quotes. |
|  | Expectations | Many patients expressed their expectations regarding their (functional) ability after the ED visits. This includes a process of coping and acceptance, for example regarding the impact on their life in general and quality of life after the ED and worries regarding these concepts. | *“When you are 40 you have a very different body than you have at my age. I notice for the first time that I am an older person. Normally I am very fit, and now I have all kinds of complaints. I do not feel like myself and that is disappointing and frustrating.”* (I 12)  *“Every time it gets worse: you do not trust your own body anymore. It is all different than what we had planned.”* (I 5) |
| General experience | Positive experience | General positive experiences that contributed to the overall experience of the patient. | *“Perfect care at (the ED of VUmc), I can recommend it to everyone. I do not think there is any better in the Netherlands. I love it, I am a very enthusiastic advertiser of the ED at VUmc. ”* (I 10)  *“I was happy to be there, because you are in good hands. My first impression was: what a nice ambiance here, very relaxing. It was a warm welcome.”* (I 9) |
|  | Negative experience | General negative experiences that contributed to the overall experience of the patient. | *“(The ED) is very un-personal, horrible, cold… I do not like it. And all the surprises they pull on me, purely unnecessary and irrational. It makes me think: what am I (swear word) doing here? It was a very disappointing ED visit.”* (I 1)  *“the coffee is too disgusting to drink, so to speak. There is no possibility to buy a sandwich or anything, while you are there the whole day. No, I got absolutely nothing (to eat).”* (I 3) |
|  | Improvement initiatives | Potential improvement initiatives that could increase patient satisfaction in the future, as voiced by the interviewed patients. | *“Of course (the nurses) come in to check on you, but just come in for a chat every now and then: they know I am in this room all by myself”* (I7).  *“Just send a physician to our house who can perform medical examinations on the spot, because there are endless examples of which you go to the ED unnecessarily”* (Wife of patient in I 5). |
